# Correction: Generative AI Models in Time-Varying Biomedical Data: Scoping Review

**DOI:** 10.2196/79605

**Published:** 2025-07-25

**Authors:** Rosemary He, Varuni Sarwal, Xinru Qiu, Yongwen Zhuang, Le Zhang, Yue Liu, Jeffrey Chiang

**Affiliations:** 1 Department of Computer Science University of California, Los Angeles Los Angeles, CA United States; 2 Department of Computational Medicine University of California, Los Angeles Los Angeles, CA United States; 3 Division of Biomedical Sciences School of Medicine University of California Riverside Riverside, CA United States; 4 Department of Biostatistics University of Michigan Ann Arbor, MI United States; 5 Institute for Integrative Genome Biology University of California Riverside Riverside, CA United States; 6 Institute for Cellular and Molecular Biology University of Texas at Austin Austin, TX United States; 7 Department of Neurosurgery David Geffen School of Medicine University of California, Los Angeles Los Angeles, CA United States

In “Generative AI Models in Time-Varying Biomedical Data: Scoping Review” (J Med Internet Res 2025;27:e59792) the authors noted one error.

In Figure 3, the right-hand category was incorrectly labeled as “Foundation models” when it should have been categorized as “Generative models.” The figure has been corrected to properly reflect this categorization, with the models listed under GANs, VAEs, Conditional VAE, DDPMs, omicsGAN, PRESCIENT, Gene-SGAN, T-GAN-D, and TimeGPT now correctly categorized under “Generative models.”

The correction will appear in the online version of the paper on the JMIR Publications website, together with the publication of this correction notice. Because this was made after submission to PubMed, PubMed Central, and other full-text repositories, the corrected article has also been resubmitted to those repositories.

Updated figure:

**Figure 3:**
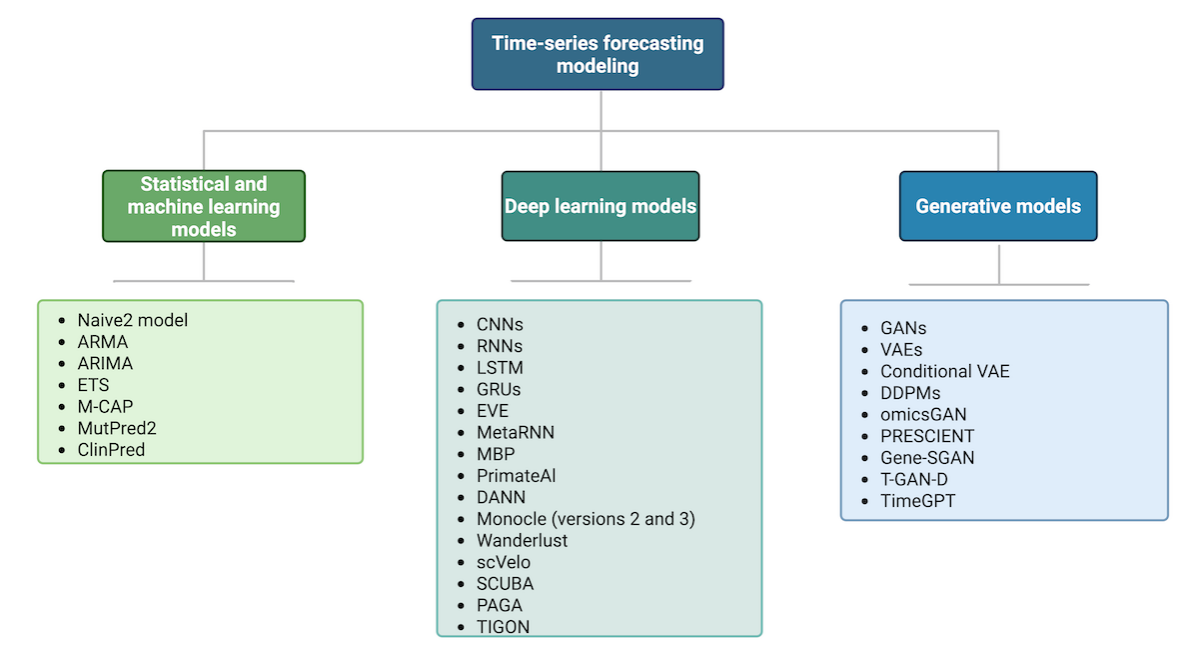
Existing models for time-series forecasting. ARIMA: autoregressive integrated moving average; ARMA: autoregressive moving average; CNN: convolutional neural network; DANN: deleterious annotation of genetic variants using neural networks; DDPM: denoising diffusion probabilistic model; ETS: exponential smoothing; EVE: Evolutionary Model of Variant Effect; GAN: generative adversarial network; GenAI: generative artificial intelligence; Gene-SGAN: gene-guided weakly supervised clustering via GANs; GRU: gated recurrent unit; LSTM: long short-term memory; M-CAP: Mendelian Clinically Applicable Pathogenicity; MBP: masked bidirectional prediction; PAGA: partition-based graph abstraction; PRESCIENT: Potential Energy Underlying Single-Cell Gradients; RNN: recurrent neural network; SCUBA: single-cell clustering using bifurcation analysis; T-GAN-D: Trained GAN Discriminator; TIGON: Trajectory Inference With Growth via Optimal Transport and Neural Network; VAE: variational autoencoder.

